# Single-Event Multi-Level Surgery in Cerebral Palsy: A Bibliometric Analysis

**DOI:** 10.3390/medicina59111922

**Published:** 2023-10-30

**Authors:** Norine Ma, Daniel Gould, Carlo Camathias, Kerr Graham, Erich Rutz

**Affiliations:** 1 Department of Paediatrics, The University of Melbourne, Melbourne, VIC 3010, Australia; 2 Medical Faculty, University of Basel, 4001 Basel, Switzerland; 3 Praxis Zeppelin, Brauerstrasse 95, 9016 St. Gallen, Switzerland; 4 Paediatric Orthopaedic Department, The Royal Children’s Hospital, Parkville, VIC 3052, Australia; 5 Hugh Williamson Gait Analysis Laboratory, The Royal Children’s Hospital, Parkville, VIC 3052, Australia; 6 Murdoch Children’s Research Institute, Melbourne, VIC 3052, Australia

**Keywords:** cerebral palsy, single-event multi-level surgery, gait analysis

## Abstract

*Background and Objectives:* Single-Event Multi-Level Surgery (SEMLS) is a complex surgical programme in which soft tissue contractures and bony torsional deformities at the ankle, knee and hip, in both lower limbs are surgically corrected during a single operative session, requiring one hospital admission and one period of rehabilitation. The aim of SEMLS is to improve gait and function in ambulant children with cerebral palsy. Utilisation of the SEMLS concept can reduce the number of surgical events, hospital inpatient stays and reduce rehabilitation requirements to a single intensive episode. Three-dimensional gait analysis is a pre-requisite to plan intervention at multiple anatomic levels to correct fixed deformities and to improve gait and function. *Materials and Methods*: This study was a bibliometric analysis of SEMLS in cerebral palsy using the Clarivate Web of Science Core Collection database from 1900 to 29 May 2023. *Results*: A total of 84 studies met the inclusion criteria. The most highly cited article was “Correction of severe crouch gait in patients with spastic diplegia with use of multilevel orthopaedic surgery” by Rodda et al. (2006) with 141 citations. The most productive institutions by number of articles were the Royal Children’s Hospital Melbourne (Australia), Murdoch Children’s Research Institute (Australia) and University of Melbourne (Australia). The most productive author by number of citations was HK Graham (Australia). *Conclusions*: The literature base for SEMLS consists largely of retrospective cohort studies. The aforementioned three institutes in Melbourne, Australia, which frequently collaborate together, have contributed the greatest number of studies in this field.

## 1. Introduction

Cerebral palsy (CP) is defined by a non-progressive injury to the brain in early development that leads to motor impairments that can impact on function [1]. Cerebral palsy is the most common cause of the upper motor neuron (UMN) syndrome in children and is characterised by a complex combination of both positive and negative features. The positive features include hypertonia, increase reflexes and clonus. The negative features include weakness, impaired balance and loss of selective motor control [2]. While there are usually no musculoskeletal deformities at birth, contractures, torsional deformities in long bones and deformities in the feet develop over time and with growth. Function of children with CP can be classified according to the topographical distribution of limb involvement—unilateral or bilateral [3]. Function is classified using the Gross Motor Function Classification System (GMFCS), with GMFCS levels I–III indicating an ambulatory patient, while GMFCS levels IV–V are non-ambulatory [4]. Cerebral palsy can also be described as spastic, dyskinetic or ataxic. The largest group of children who benefit from SEMLS have a spastic motor disorder [5].

**Figure 1:**
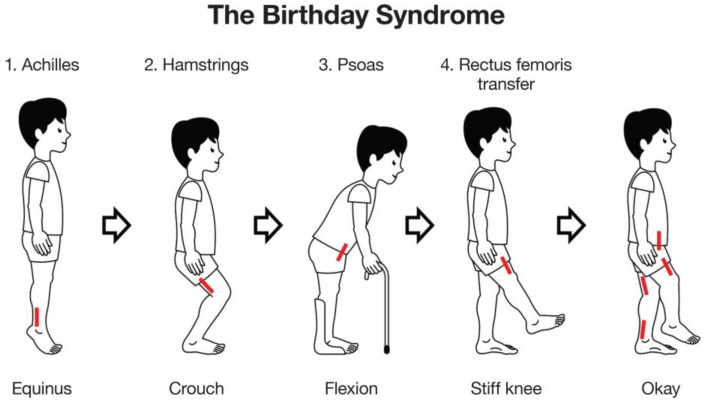
The Birthday Syndrome. Many children with cerebral palsy had multiple episodes of surgery for lower limb deformities during childhood to improve gait and function. Surgery often started distally with lengthening of the Achilles’ tendons for equinus gait or “toe-walking” (1 Achilles) [15]. Flexed knee or crouch gait was corrected two to three years later by hamstring lengthening (2 hamstrings), followed later still by lengthening of the psoas for hip flexion and rectus femoris transfer for “stiff-knee gait” (3 psoas, 4 rectus femoris transfer). Many children would spend several birthdays having surgery or recovering from surgery [9]. In essence this is single level, multi-event surgery. NB: the approximate site for each surgery is indicated by the red lines.

**Figure 2:**
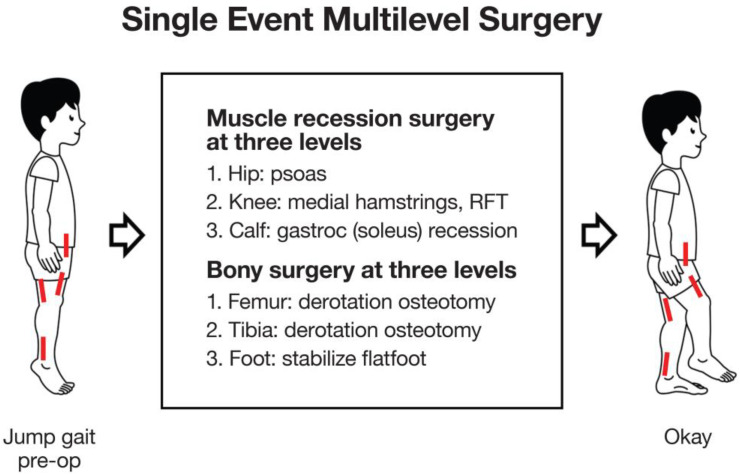
Single-Event, Multi-level Surgery (SEMLS): Sagittal plane/soft tissue surgery. SEMLS is the acronym for “Single Event, Multilevel Surgery” the concept of identifying and correcting all clinically relevant, musculoskeletal deformities, bony and soft tissue, during one surgical session. This illustration shows only the three levels of soft tissue surgery (short red lines), for contracted muscle-tendon units, which work mainly in the sagittal plane. NB: Jump gait is the most common gait pattern in children with diplegia and is characterized by spastic contractures at the hip, knee and ankle levels [16]. RFT = rectus femoris transfer.

Musculoskeletal pathology can impact on function by resulting in increased difficulty with walking, participation in family life, schooling and participation in the community. Some contractures and some deformities such as deformities of the feet can be painful and limit choice of footwear [6]. Children are assessed for management of these musculoskeletal impairments using a variety of tools ranging from clinical examination and imaging to three-dimensional gait analysis (3DGA) [7]. The combination of 3DGA, physical examination and radiology is referred to as the “diagnostic matrix”. Management may include physical therapy, casting, orthoses, management of spasticity using botulinum toxin A injections or neurosurgical procedures such as selective dorsal rhizotomy (SDR) and ultimately, orthopaedic surgery for the correction of fixed deformities. Prior to the concept of single-event multi-level surgery (SEMLS), surgeons would address musculoskeletal abnormalities with sequential surgeries. This led to the term “birthday syndrome” introduced by Dr Mercer Rang due to children with CP undergoing isolated surgical procedures each year, followed by long periods of rehabilitation (Figure 1) [8]. After one pathology was treated and post-operative recovery was complete, further impairments would become evident leading to the need for subsequent surgery. SEMLS aims to correct multiple soft tissue and/or bony deformities during one operation, leading to a reduced number of surgical admissions and one period of intense rehabilitation (Figure 2). Improvement in gait parameters after SEMLS have been well documented in the literature [9,10]. Many children have a reduced need for assistive devices with improvements on the Functional Mobility Scale (FMS) and some will have improvements in gross motor function according to the Gross Motor Function Measure (GMFM) [11,12]. SEMLS has also been shown to improve children’s quality of life, particularly the child’s perceived ability to walk and feelings about functioning [13,14].

**Figure 3:**
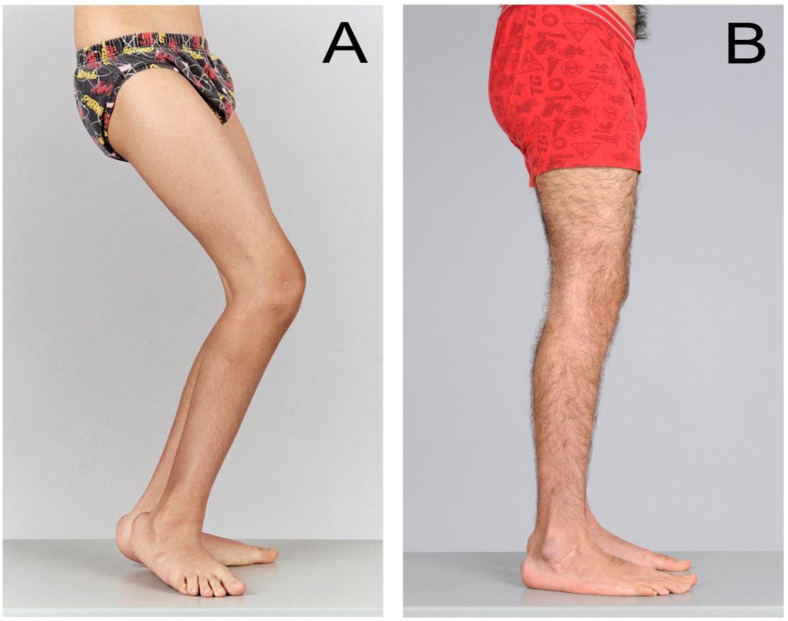
(**A**,**B**) SEMLS surgery: soft tissue and sagittal plane correction. These are photographs of a 10-year-old boy with spastic diplegia, GMFCS Level III, (**A**) before and (**B**) 8 years after SEMLS which included bilateral gastrocsoleus recession, hamstring lengthening/transfer and recession of the iliopsoas. At 8-year follow up, he is skeletally mature and all deformities remain corrected. The hips and knees extend fully and the feet are plantigrade. Pre- and post-operative evaluation in the Gait Laboratory confirmed clinically and statistically significant improvements in gait and gross motor function. No revision or repeat surgery was required.

**Figure 4:**
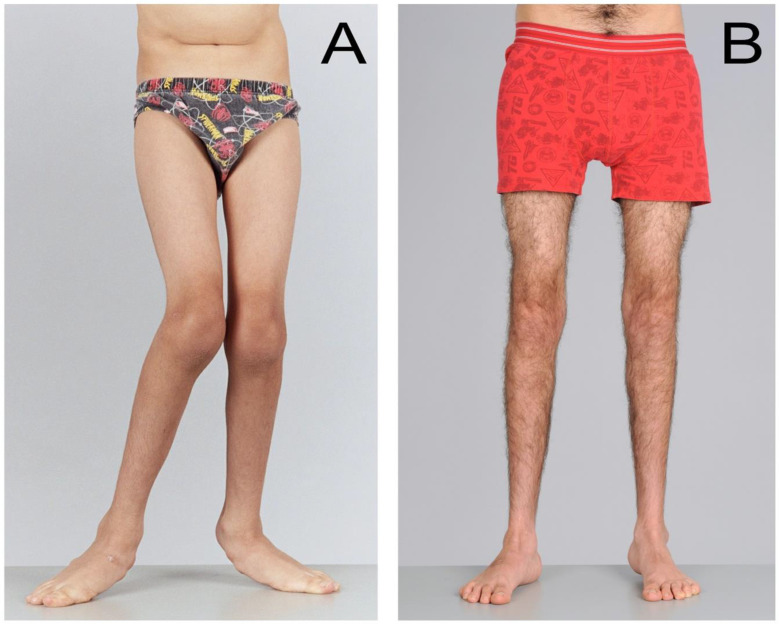
(**A**,**B**) SEMLS surgery: bony tissue and transverse plane correction. These are the frontal or coronal plane photographs of the same 10-year-old boy with spastic diplegia, as seen in Figure 3A,B. Pre-operatively, he had bilateral medial femoral torsion (aka femoral anteversion) noted by the “squinting knee caps”, bilateral external tibial torsion and bilateral pes valgus (flat feet). The bony surgery consisted of bilateral external rotational osteotomies of the femur, internal rotation osteotomies of the tibiae and stabilization of the flat feet by bone grafting. All deformities remained fully corrected at 8-year follow up, with no additional surgery.

Soft tissue procedures such as psoas, hip adductor, hamstring or calf muscle lengthening aim to counteract the effects of contractures and can improve crouch gait or equinus gait (Figure 3). Bony procedures such as rotational osteotomies aim to improve limb alignment and joint congruity, and contribute to the management of hip dysplasia (Figure 4). However, surgical management at one anatomic level can have consequences at other levels. For example, correction of equinus deformity by performing isolated calf lengthening surgery at the incorrect dose can lead to crouch gait [17]. A multidisciplinary approach is important to guide decision making for management of these musculoskeletal impairments and optimise post-operative recovery including contributions from physicians, biomedical engineers, physiotherapists, orthotists, occupational therapists and social workers in addition to surgeons.

This study is a bibliometric analysis which aims to provide an overview of the state of the literature base in SEMLS by identifying trends in publication output on the topic of SEMLS from 1990 to 2023, identifying the most productive authors, countries and institutions in the field, and evaluating the quality of studies.

## 2. Materials and Methods

The Clarivate Web of Science Core Collection database was searched on 29 May 2023 using the keywords “cerebral palsy” and “multi-level surgery” or “SEMLS” in all fields, from 1900 to 29 May 2023. A single database was used to ensure consistency of citation data across included studies, acknowledging that some journals may not be indexed in the Web of Science. The Core Collection only was used to ensure all studies were accompanied with citation data. All original studies or reviews of SEMLS in cerebral palsy with predominantly paediatric patients were included (Table 1). Studies of upper limb surgery or only one surgical technique as part of SEMLS were excluded. Two independent reviewers screened articles for inclusion. Clarivate Journal Citation Reports were searched for the 2021 impact factors for included journals where available. The 2014 impact factor was used for The Journal of Bone and Joint Surgery—British Volume due to continuation of the journal as the Bone and Joint Journal. The Journal Citation Indicator (JCI) was also extracted from Clarivate Journal Citation Reports. This is a newer metric which controls for subject category, uses a three-year period (instead of the two-year period used for the journal impact factor), and includes all citations from publication of an article. A JCI of 1.0 represents the average which is compared to a global baseline. As SEMLS is a field which has relatively few papers, this may be able to provide a more accurate representation of a journal’s impact in this field as they can be compared against other journals within similar subject categories. The collaboration index [18] was calculated for each year, as well as across all years.

The author characteristic index (c-index) was calculated for the top nine most productive authors. This assigns weighting for an author’s position in the authorship of each publication, where the first author and last author of a publication are given more weight [19].

Seventy-two observational studies were assessed using the Methodological Index for Non-randomised Studies (MINORS) [20]. A score out of 18 was given to a cohort study based on study design including inclusion criteria, retrospective or prospective collection of data, blinding of endpoints, follow-up period and loss to follow-up. A score of less than 10 was considered low quality evidence. A score between 10 and 14 (inclusive) was considered fair quality, and a score over 15 was considered good quality evidence.

Network visualisations were constructed using VOSViewer version 1.6.19 from data collected from the Clarivate Web of Science Core Collection. Bibliographic coupling by authors determines connectiveness based on similarity of reference lists between publications by each author. Co-occurrence of keywords indicates the most commonly used keywords within the documents studied. Visualisation of the citation analysis indicates the connectivity of each article based on number of times they cite each other. This allows visualisation of the relationship between authors that publish frequently in this field, as well as provides a visualisation of the common concepts studied within SEMLS.

## 3. Results

A total of 84 studies were included in the bibliometric analysis (Table S1). Study types included 6 review articles, 61 retrospective studies, 15 prospective studies, 1 cost analysis and 1 randomised controlled trial. Sample sizes ranged from 6 to 1715 limbs. Studies included both ambulatory and non-ambulatory patients (across all GMFCS levels). Most studies investigated bilateral CP, or a mix of unilateral and bilateral, although a few studies focused solely on unilateral CP.

The three most highly cited articles were Rodda 2006 [21], McGinley 2012 [22] and Thomason 2013 [11], with 141, 127 and 112 citations, respectively. These were all affiliated with the Royal Children’s Hospital Melbourne, Australia.

**Figure 5:**
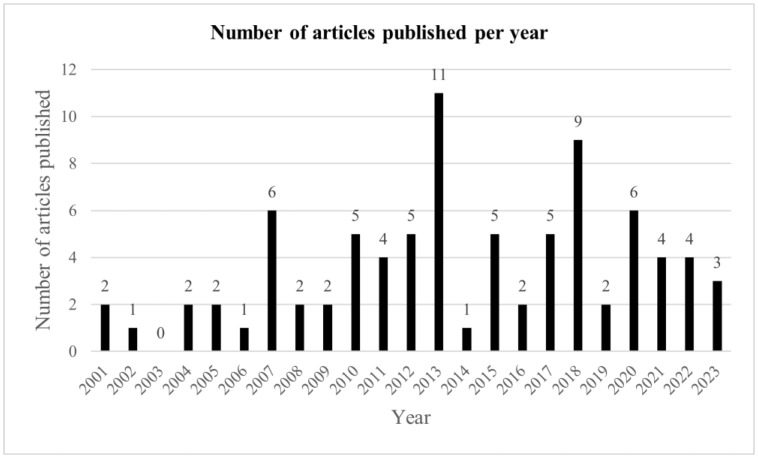
Number of studies by year.

Study dates ranged from 2001 to 2023 inclusive (Figure 5). The greatest number of studies were published in 2013 (11 papers).

**Figure 6:**
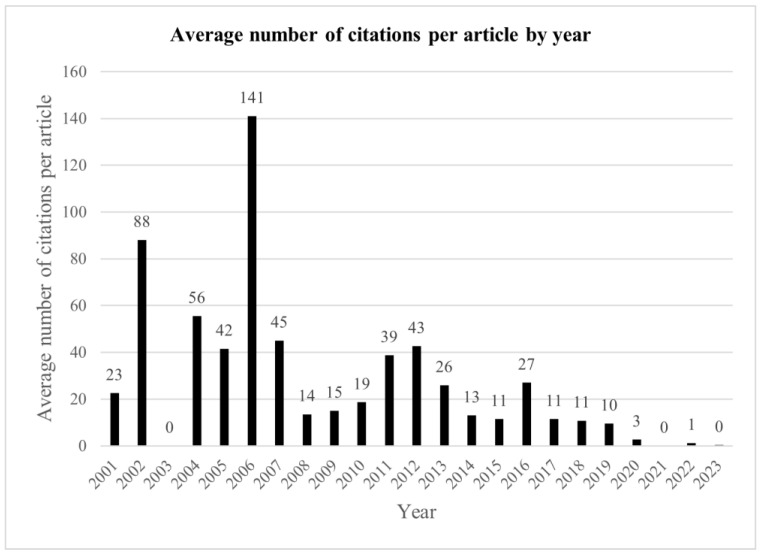
Average number of citations per article by year.

**Figure 7:**
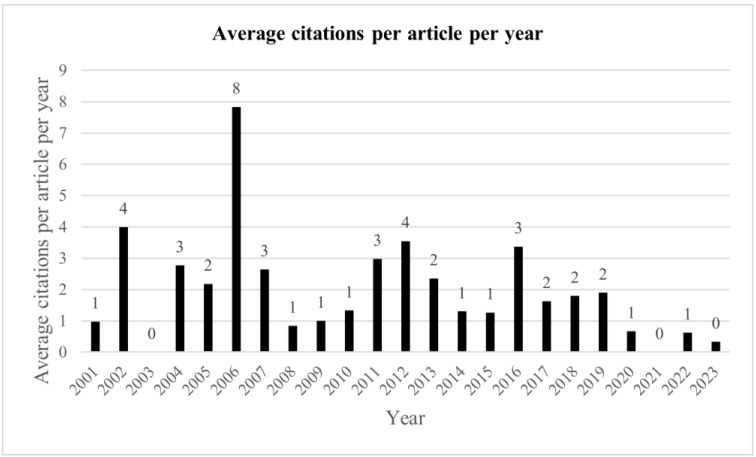
Average number of citations per article by year, divided by number of years from 2023.

The number of citations per article trended lower in more recent years, with the highly cited articles by Rodda 2006 [21] and Saraph 2002 [23] causing a spike in the number of citations in their respective years (Figure 6). When corrected for number of years since publication, the number of citations per article remains more static; however, again Rodda 2006 stands out due to the high number of citations (Figure 7).

The most productive country by number of publications was the United States of America (USA) with 19 publications, followed by Australia and Austria (Table 2).

The most productive author by number of publications was Graham HK with 12 publications, 782 total citations and 65 average citations per publication (Table 3). This includes all positions of authorship.

The three most productive institutions by number of publications were all from Melbourne, Australia and were frequent collaborators with each other (Table 4). 

Studies included were published in 37 unique journals (Table 5). The journal with the highest number of SEMLS publications was Gait and Posture which published 13 articles with a total of 134 citations. The journal with the most citations per article was Acta Orthopaedica (UK), which published two articles with an average of 36 citations per article. Impact factors were not able to be obtained for all journals; however, those that were able to be obtained ranged from 0.831 (AORN Journal) to 7.892 (Biomedical Journal). Only 13 of the 37 journals had a JCI higher than the average of 1.0. However, each journal had a unique combination of subject categories, impacting comparison of the JCI between them. The three most common subject categories were ‘orthopedics’, ‘pediatrics’ and ‘surgery’; however, not all journals included in this study were in one of these three subject categories. In general, a higher impact factor did correlate with a higher JCI.

The median MINORS score for the 72 observational studies assessed was 10, indicating most studies were graded as “fair quality”. Most studies were retrospective and unblinded, without prospective sample size calculation.

The collaboration index across all years was 5.5 and ranged from 3.5 to 8.0. The collaboration index increased in recent years, with the collaboration index of papers published in 2023 being 8.0. Number of authors per paper ranged from 1 to 11, with a median of 5 authors per paper (19 studies had 5 authors).

**Figure 8:**
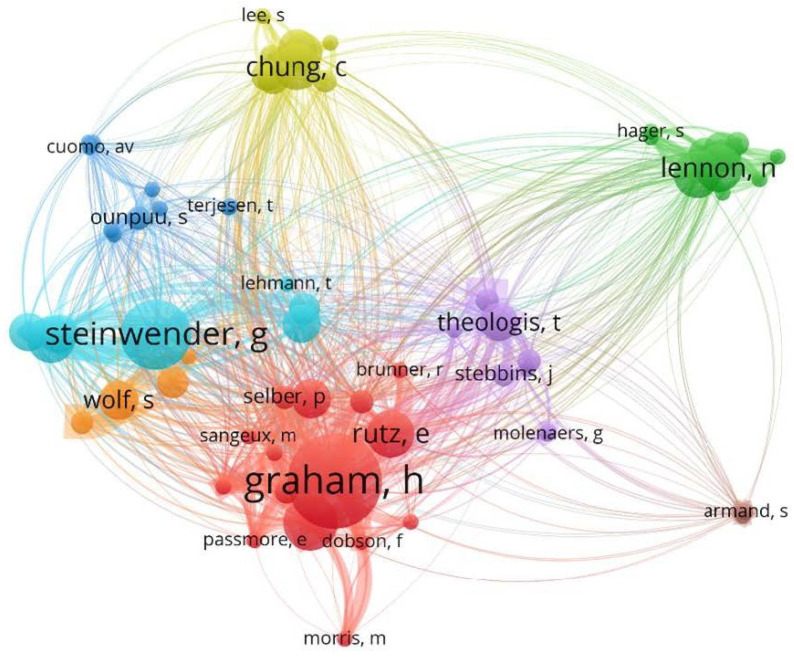
Bibliographic coupling by authors, minimum 2 connections.

Bibliographic coupling by authors (Figure 8) shows overlap of reference lists between publications by authors. The visualisation suggests that authors from the same institution tend to cite a similar set of papers as each network group tends to include authors from the same institution.

**Figure 9:**
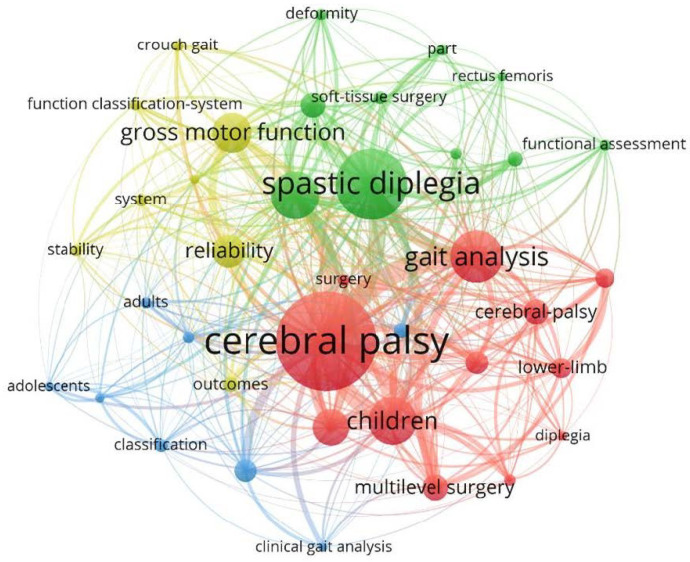
Co-occurrence of keywords.

The three most common keywords used were ‘cerebral palsy’, ‘gait analysis’ and ‘spastic diplegia’, giving an indication of the subset of CP usually undergoing SEMLS (Figure 9). Despite knowing that surgical intervention affects children with unilateral and bilateral cerebral palsy differently, many studies did not specify the topographical classification of their sample patients or GMFCS levels. 

## 4. Discussion

There has been growing interest in the study of SEMLS in CP since 2001, with a wide range of institutions and countries contributing to the evidence base [22]. Major contributors to the literature include the Royal Children’s Hospital Melbourne (Australia), Murdoch Children’s Research Institute (Australia) and The University of Melbourne (Australia), with three major landmark papers which continue to be highly cited to this date—“Correction of severe crouch gait in patients with spastic diplegia with use of multilevel orthopaedic surgery” by Rodda et al. (2006), “Single-event multilevel surgery for children with cerebral palsy: a systematic review” by McGinley et al. (2012) and “Single-Event Multilevel Surgery in Children with Spastic Diplegia A Pilot Randomized Controlled Trial” by Thomason et al. (2011). These institutions work closely together and utilise the Hugh Williamson Gait Analysis Laboratory, opened in 1995, with teams built over the subsequent five years including surgery, orthotics and rehabilitation. The study by Thomason et al. [12] was the first RCT on SEMLS. Due to ethical considerations and limitation of resources, it takes substantial planning, funding and research resources to conduct an RCT in SEMLS. This may explain the large number of uncontrolled cohort studies that predominate the field. The Thomason RCT overcame ethical concerns by carefully constructed inclusion criteria, offering control patients an effective alternative, Progressive Resistance Strength Training (PRST), and facilitating crossover to the SEMLS surgery after 12 months. This study provides high-quality evidence of improvement in gait in children with bilateral cerebral palsy 12 months after SEMLS, with a 34% improvement in GPS and 57% improvement in GGI. 

The most productive author by number of citations, HK Graham, is also affiliated with the aforementioned institutions. Of note, the authors that published more articles by quantity did not necessarily correspond with the highest number of total citations. These authors would have a higher h-index, which is an index used to compare the impact of authors [24]. However, it is better used to compare authors at similar career stages, as an author with a large number of publications with lower number of citations may have a higher h-index than an author with a small number of highly cited articles. The g-index has been proposed as a way to take this into account, which is calculated similarly to the h-index except it uses the highest number of papers that have received g2 or more citations [25]. Furthermore, another factor that may affect the perceived impact of an author is the amount of collaboration one partakes in. It is assumed that the first and last author of an article have likely made more substantial contributions to the research than the other authors. This is taken into account with calculation of the c-index where more weighting is given to first or last authorship [26]. Additionally, if an article has more authors, each author has likely contributed less to the project. The A-index was developed to take this into account which can then be used to calculate other indices of collaboration and impact [27].

The most productive journal in the field of SEMLS was Gait and Posture. Interestingly, this is different to the most productive journal in hip surgery in cerebral palsy, which is the Journal of Pediatric Orthopaedics (USA) [27]. These two journals have similar impact factors of 2.746 and 2.537, respectively. This difference of preference for journal of publication in these two fields within cerebral palsy research may be due to the scope of the journals or author preference. 

The keyword analysis shows that most studies included patients with bilateral CP and specified this in their keywords. This indicates consistency in the use of the term as well as that SEMLS is typically indicated in patients with bilateral CP. Although there are efforts to shift away from the use of descriptors such as hemiplegia, diplegia or quadriplegia, these remain as the most commonly used descriptors in the current evidence base [1]. One of the more common keywords highlighted in the keyword analysis are “gross motor function” and “function classification-system”. The GMFCS is an important way to classify the functional status of children with CP as it allows consistency between studies when comparing inclusion criteria. “Gait analysis” is also a commonly used keyword. Three-dimensional gait analysis has become the gold standard in objectively assessing gait parameters to guide decision making for SEMLS and allows the measurement of change in these parameters after surgery [28]. Parameters measured can include temporospatial parameters such as cadence, stride length and walking velocity, as well as kinematics in three planes [29]. Gait kinematics can be combined to form a summary statistic of gait that provides an indication of variance from neurotypical gait, with several such scores having been developed. The Gait Deviation Index (GDI) combines 15 gait features to provide an indication of functional walking ability, with a GDI greater than or equal to 100 indicating absence of gait pathology [30]. The Gait Variable Score (GVS) describes the difference of a single gait variable from typical and can be combined to form a Movement Analysis Profile (MAP). The Gait Profile Score (GPS) indicates the difference from normal gait across nine kinematic variables [31]. These gait parameters can be assessed pre- and post-SEMLS to understand the effect of these surgeries on specific joints, or overall gait. However, as SEMLS is catered specifically to each patient’s profile of musculoskeletal characteristics, it can be difficult to compare outcomes as a wide variety of combinations of surgeries are included for each patient. Hence, many studies focus on one specific surgery in the context of SEMLS. This does not take into account the effects that surgery at one level may have on another level [32]. In 2013, it was noted that there was a large spike in the number of articles published compared to other years. These articles were published by a range of institutions. Several of these studies were long-term follow-up studies of SEMLS. This indicates that this may coincide with a time period where there was an increase in the availability and popularity of SEMLS several years prior, allowing multiple institutions to complete long-term follow-up studies.

The current literature base in SEMLS is predominantly composed of retrospective cohort studies of variable quality, meaning conclusions are limited by bias and confounding factors [22]. The median MINORS score of ten indicates that there are still many areas for improvement in SEMLS study methodology. While prospective studies with comparator groups and RCTs are logistically more difficult to conduct and require more planning and time to conduct, they provide a higher level of evidence and reduce bias and confounding factors. This is particularly of importance as many studies of CP have small sample sizes due to few children meeting study inclusion criteria. However, due to the progressive nature of musculoskeletal pathology in CP, ethical considerations prevent the design of randomised control groups that do not receive surgical intervention for more than 12 months. Most studies to date have been retrospective, but more focus should be put into designing appropriate prospective studies to improve the overall quality of the evidence base. Multicentre studies are also required to standardise SEMLS, rehabilitation and increase power and transparency.

## 5. Conclusions

The current literature in SEMLS for children with cerebral palsy is predominantly composed of fair quality retrospective cohort studies. The journal Gait and Posture has published the highest quantity of studies in the field. The most influential studies by number of citations have been affiliated with the Royal Children’s Hospital Melbourne, Murdoch Children’s Research Institute and The University of Melbourne, all Australian institutions. The most influential author by number of citations was HK Graham, affiliated with the aforementioned Australian institutions.

## Figures and Tables

**Table 1:** Inclusion and exclusion criteria for articles included in this study.

Inclusion Criteria	Exclusion Criteria
SEMLS defined as a minimum of two surgical procedures at two different anatomic levels (i.e., hip, knee or ankle)	Case studies, case series or conference abstracts
Studies including children with cerebral palsy	Studies of only one surgical technique as part of SEMLS
Studies of outcomes of SEMLS	>50% adult patients included
Original studies or reviews	Studies of upper limb surgery

**Table 2:** Top five most productive countries by number of publications.

Country	No. of Publications
United States of America	19
Australia	15
Austria	10
UK	9
Switzerland	9

**Table 3:** Top nine most productive authors by number of publications.

Author	No. of Publications	Total Citations	Average Citations per Publication	c-Index
Graham HK	12	782	65	10.0
Steinwender G	9	271	30	5.3
Baker R	7	446	64	4.0
Rutz E	6	250	42	4.0
Zwick EB	6	214	36	3.3
Chung CY	6	82	14	4.5
Park MS	6	82	14	4.7
Lennon N	6	43	7	5.5
Miller F	6	43	7	3.8

**Table 4:** Top six most productive institutions by number of publications.

Affiliations	Country	No. of Publications
Royal Children’s Hospital Melbourne	Australia	14
Murdoch Children’s Research Institute	Australia	10
University of Melbourne	Australia	10
Medical University of Graz	Austria	6
Seoul National University	South Korea	6
Ruprecht Karls University Heidelberg	Germany	5

**Table 5:** List of included journals by number of articles published. * not available.

Journal	Country	No. of Articles	No. of Citations	Average No. of Citations per Article	Impact Factor (2021)	JCI
Gait and Posture	International	13	134	10.3	2.746	0.84
Journal of Pediatric Orthopaedics	USA	12	214	17.8	2.537	0.98
Developmental Medicine and Child Neurology	UK	9	91	10.1	4.864	1.43
Journal of Bone and Joint Surgery-British Volume	UK	5	81	16.2	3.309	*
Journal of Bone and Joint Surgery-American Volume	USA	3	0	0.0	6.558	2.21
Journal of Childrens Orthopaedics	Germany	3	26	8.7	1.917	0.63
Bone and Joint Journal	UK	3	106	35.3	5.385	1.92
Clinical Orthopaedics and Related Research	USA	2	42	21.0	4.837	1.72
Acta Orthopaedica	UK	2	72	36.0	3.925	1.34
Indian Journal of Orthopaedics	Germany	2	53	26.5	1.038	0.38
Pediatric Physical Therapy	USA	2	44	22.0	1.466	0.56
Research in Developmental Disabilities	USA	2	33	16.5	3	1.46
Eklem Hastaliklari Ve Cerrahisi-Joint Diseases and Related Surgery	Turkey	2	26	13.0	1.338	*
PLoS ONE	USA	1	0	0.0	3.752	0.88
Journal of Rehabilitation Medicine	Sweden	1	0	0.0	3.959	1.03
Human Movement Science	Netherlands	1	0	0.0	2.397	0.7
Pediatrics	USA	1	0	0.0	9.703	3.17
Disability and Rehabilitation	UK	1	0	0.0	2.439	1.08
Biomedical Journal	Netherlands	1	31	31.0	7.892	1.18
Scientific Reports	UK	1	23	23.0	4.997	1.05
European Journal of Paediatric Neurology	UK	1	20	20.0	3.692	1.04
Zeitschrift Fur Orthopadie Und Ihre Grenzgebiete	Germany	1	19	19.0	1.108	0.35
Journal of Pediatric Orthopaedics-Part B	USA	1	17	17.0	1.473	0.49
JBJS Reviews	USA	1	17	17.0	*	0.55
Archives of Orthopaedic and Trauma Surgery	Germany	1	14	14.0	2.928	1.08
Clinical Biomechanics	UK	1	14	14.0	2.034	0.65
Journal of Foot and Ankle Surgery	USA	1	13	13.0	1.345	0.46
Journal of Orthopaedics	India	1	13	13.0	*	0.62
Journal of Orthopaedics Trauma and Rehabilitation	Netherlands	1	12	12.0	*	0.11
Journal of Child Neurology	USA	1	11	11.0	2.363	0.66
AORN Journal	USA	1	10	10.0	0.831	0.34
Trauma-Spain	Spain	1	9	9.0	*	*
Childs Nervous System	Germany	1	8	8.0	1.532	0.48
Frontiers in Neuroscience	Switzerland	1	8	8.0	5.152	0.87
Medicine	USA	1	7	7.0	1.817	0.39
Current Orthopaedic Practice	USA	1	6	6.0	*	0.1
Journal of Evolution of Medical And Dental Sciences-JEMDS	India	1	4	4.0	*	0.02

## Data Availability

The data presented in this study are available in Table S1.

## Supplementary Materials

The following supporting information can be downloaded at https://www.mdpi.com/article/10.3390/medicina59111922/s1, Table S1: List of included studies.

## References

[1] Rosenbaum P., Paneth N., Leviton A., Goldstein M., Bax M., Damiano D., Dan B., Jacobsson B. (2007). A report: The definition and classification of cerebral palsy April 2006. Dev. Med. Child Neurol. Suppl..

[2] Panteliadis C.P. (2018). Cerebral Palsy: A multidisciplinary Approach.

[3] Andersen G.L., Irgens L.M., Haagaas I., Skranes J.S., Meberg A.E., Vik T. (2008). Cerebral palsy in Norway: Prevalence, subtypes and severity. Eur. J. Paediatr. Neurol..

[4] Palisano R.J., Rosenbaum P., Bartlett D., Livingston M.H. (2008). Content validity of the expanded and revised Gross Motor Function Classification System. Dev. Med. Child Neurol..

[5] Graham H.K., Rosenbaum P., Paneth N., Dan B., Lin J.-P., Damiano D.L., Becher J.G., Gaebler-Spira D., Colver A., Reddihough D.S. (2016). Cerebral palsy. Nat. Rev. Dis. Primers.

[6] Jung N.H., Pereira B., Nehring I., Brix O., Bernius P., Schroeder S.A., Kluger G.J., Koehler T., Beyerlein A., Weir S. (2014). Does hip displacement influence health-related quality of life in children with cerebral palsy?. Dev. Neurorehabilit..

[7] Shelly A., Davis E., Waters E., Mackinnon A., Reddihough D., Boyd R., Reid S., Graham H.K. (2008). The relationship between quality of life and functioning for children with cerebral palsy. Dev. Med. Child Neurol..

[8] Davids J.R., Õunpuu S., DeLuca P.A., Davis R.B. (2003). Optimization of Walking Ability of Children with Cerebral Palsy. JBJS.

[9] Rang M., Weinstein S.L., Flynn J.M. (1990). Cerebral palsy. Lovell and Winter’s Pediatric Orthopaedics.

[10] Gough M., Eve L.C., Robinson R.O., Shortland A.P. (2004). Short-term outcome of multilevel surgical intervention in spastic diplegic cerebral palsy compared with the natural history. Dev. Med. Child Neurol..

[11] Thomason P., Selber P., Graham H.K. (2013). Single Event Multilevel Surgery in children with bilateral spastic cerebral palsy: A 5 year prospective cohort study. Gait Posture.

[12] Thomason P., Baker R., Dodd K., Taylor N., Selber P., Wolfe R., Graham H.K. (2011). Single-event multilevel surgery in children with spastic diplegia: A pilot randomized controlled trial. JBJS.

[13] Schranz C., Kruse A., Kraus T., Steinwender G., Svehlik M. (2017). Does unilateral single-event multilevel surgery improve gait in children with spastic hemiplegia? A retrospective analysis of a long-term follow-up. Gait Posture.

[14] Himpens E., Franki I., Geerts D., Tack R., Van Der Looven R., Van den Broeck C. (2013). Quality of life in youngsters with cerebral palsy after single-event multilevel surgery. Eur. J. Paediatr. Neurol..

[15] Borton D., Walker K., Pirpiris M., Nattrass G., Graham H. (2001). Isolated calf lengthening in cerebral palsy: Outcome analysis of risk factors. J. Bone Jt. Surg. Br. Vol..

[16] Sutherland D.H., Davids J.R. (1993). Common gait abnormalities of the knee in cerebral palsy. Clin. Orthop. Relat. Res..

[17] Firth G.B., Passmore E., Sangeux M., Thomason P., Rodda J., Donath S., Selber P., Graham H.K. (2013). Multilevel surgery for equinus gait in children with spastic diplegic cerebral palsy: Medium-term follow-up with gait analysis. JBJS.

[18] Lawani S.M. (1980). Quality, Collaboration and Citations in Cancer Research: A Bibliometric Study.

[19] Ciaccio E.J., Bhagat G., Lebwohl B., Lewis S.K., Ciacci C., Green P.H. (2019). Comparison of several author indices for gauging academic productivity. Inform. Med. Unlocked.

[20] Slim K., Nini E., Forestier D., Kwiatkowski F., Panis Y., Chipponi J. (2003). Methodological index for non-randomized studies (MINORS): Development and validation of a new instrument. ANZ J. Surg..

[21] Rodda J., Graham H.K., Nattrass G., Galea M.P., Baker R., Wolfe R. (2006). Correction of severe crouch gait in patients with spastic diplegia with use of multilevel orthopaedic surgery. JBJS.

[22] McGinley J.L., Dobson F., Ganeshalingam R., Shore B.J., Rutz E., Graham H.K. (2012). Single-event multilevel surgery for children with cerebral palsy: A systematic review. Dev. Med. Child Neurol..

[23] Saraph V., Zwick E.-B., Zwick G., Steinwender C., Steinwender G., Linhart W. (2002). Multilevel surgery in spastic diplegia: Evaluation by physical examination and gait analysis in 25 children. J. Pediatr. Orthop..

[24] Hirsch J.E. (2005). An index to quantify an individual’s scientific research output. Proc. Natl. Acad. Sci. USA.

[25] Egghe L. (2006). An improvement of the h-index: The g-index. ISSI Newsl..

[26] Stallings J., Vance E., Yang J., Vannier M.W., Liang J., Pang L., Dai L., Ye I., Wang G. (2013). Determining scientific impact using a collaboration index. Proc. Natl. Acad. Sci. USA.

[27] Ma N., Sclavos N., Graham K., Rutz E. (2023). Hip Surgery in Cerebral Palsy: A Bibliometric Analysis. Int. J. Environ. Res. Public Health.

[28] Ma N., Sclavos N., Passmore E., Thomason P., Graham K., Rutz E. (2021). Three-dimensional gait analysis in children undergoing gastrocsoleus lengthening for equinus secondary to cerebral palsy. Medicina.

[29] O’Byrne J.M., Jenkinson A., O’brien T. (1998). Quantitative analysis and classification of gait patterns in cerebral palsy using a three-dimensional motion analyzer. J. Child Neurol..

[30] Schwartz M.H., Rozumalski A. (2008). The Gait Deviation Index: A new comprehensive index of gait pathology. Gait Posture.

[31] Baker R., McGinley J.L., Schwartz M.H., Beynon S., Rozumalski A., Graham H.K., Tirosh O. (2009). The gait profile score and movement analysis profile. Gait Posture.

[32] Lofterød B., Terjesen T. (2008). Results of treatment when orthopaedic surgeons follow gait-analysis recommendations in children with CP. Dev. Med. Child Neurol..

