# Supplementary material for: Single-Event Multi-Level Surgery in Cerebral Palsy: A Bibliometric Analysis
Source: Medicina (Kaunas). 2023 Oct 30;59(11):1922. doi: 10.3390/medicina59111922 (PMC10672936; doi:10.3390/medicina59111922)
Supplement: Supplementary file 1 [file medicina-59-01922-s001.zip › medicina-2670729-supplementary.pdf]

Supplementary material Table S1: List of included studies

First Author	Year Published	Title	Journal	Times Cited
Rodda	2006	Correction Of Severe Crouch Gait In Patients With Spastic Diplegia With Use Of Multilevel Orthopaedic Surgery	Journal Of Bone And Joint Surgery-American Volume	141
McGinley	2012	Single-Event Multilevel Surgery For Children With Cerebral Palsy: A Systematic Review	Developmental Medicine And Child Neurology	127
Thomason	2011	Single-Event Multilevel Surgery In Children With Spastic Diplegia A Pilot Randomized Controlled Trial	Journal Of Bone And Joint Surgery-American Volume	112
Gough	2004	Short-Term Outcome Of Multilevel Surgical Intervention In Spastic Diplegic Cerebral Palsy Compared With The Natural History	Developmental Medicine And Child Neurology	88
Saraph	2002	Multilevel Surgery In Spastic Diplegia: Evaluation By Physical Examination And Gait Analysis In 25 Children	Journal Of Pediatric Orthopaedics	88
Thomason	2013	Single Event Multilevel Surgery In Children With Bilateral Spastic Cerebral Palsy: A 5 Year Prospective Cohort Study	Gait & Posture	85
Harvey	2007	The Functional Mobility Scale: Ability To Detect Change Following Single Event Multilevel Surgery	Developmental Medicine And Child Neurology	76
Adolfsen	2007	Kinematic And Kinetic Outcomes After Identical Multilevel Soft Tissue Surgery In Children With Cerebral Palsy	Journal Of Pediatric Orthopaedics	64
Cuomo	2007	Health-Related Quality Of Life Outcomes Improve After Multilevel Surgery In Ambulatory Children With Cerebral Palsy	Journal Of Pediatric Orthopaedics	63
Firth	2013	Multilevel Surgery For Equinus Gait In Children With Spastic Diplegic Cerebral Palsy	Journal Of Bone And Joint Surgery-American Volume	49
Rutz	2012	Stability Of The Gross Motor Function Classification System After Single-Event Multilevel Surgery In Children With Cerebral Palsy	Developmental Medicine And Child Neurology	43
Rutz	2013	Explaining The Variability Improvements In Gait Quality As A Result Of Single Event Multi-Level Surgery In Cerebral Palsy	Gait & Posture	42
Saraph	2005	Gait Improvement Surgery In Diplegic Children - How Long Do The Improvements Last?	Journal Of Pediatric Orthopaedics	42
Sung	2013	Long Term Outcome Of Single Event Multilevel Surgery In Spastic Diplegia With Flexed Knee Gait	Gait & Posture	41
Dobson	2005	Multilevel Orthopaedic Surgery In Group Iv Spastic Hemiplegia	Journal Of Bone And Joint Surgery-British Volume	41
Lamberts	2016	A Systematic Review Of The Effects Of Single-Event Multilevel Surgery On Gait Parameters In Children With Spastic Cerebral Palsy	Plos One	36

Steinwender	2001	Fixed And Dynamic Equinus In Cerebral Palsy: Evaluation Of Ankle Function After Multilevel Surgery	Journal Of Pediatric Orthopaedics	36
Thompson	2010	The Use Of Minimally Invasive Techniques In Multi-Level Surgery For Children With Cerebral Palsy Preliminary Results	Journal Of Bone And Joint Surgery-British Volume	34
Patikas	2007	Electromyographic Patterns In Children With Cerebral Palsy: Do They Change After Surgery?	Gait & Posture	33
Ounpuu	2015	Long-Term Outcomes After Multilevel Surgery Including Rectus Femoris, Hamstring And Gastrocnemius Procedures In Children With Cerebral Palsy	Gait & Posture	32
Dreher	2018	Long-Term Development Of Gait After Multilevel Surgery In Children With Cerebral Palsy: A Multicentre Cohort Study	Developmental Medicine And Child Neurology	31
Svehlik	2011	The Influence Of Age At Single-Event Multilevel Surgery On Outcome In Children With Cerebral Palsy Who Walk With Flexed Knee Gait	Developmental Medicine And Child Neurology	31
Godwin	2009	The Gross Motor Function Classification System For Cerebral Palsy And Single-Event Multilevel Surgery: Is There A Relationship Between Level Of Function And Intervention Over Time?	Journal Of Pediatric Orthopaedics	23
Metaxiotis	2004	Conversion Of Biarticular To Monoarticular Muscles As A Component Of Multilevel Surgery In Spastic Diplegia	Journal Of Bone And Joint Surgery-British Volume	23
Harvey	2012	Longitudinal Changes In Mobility Following Single-Event Multilevel Surgery In Ambulatory Children With Cerebral Palsy	Journal Of Rehabilitation Medicine	20
Lee	2010	Level Of Improvement Determined By Podci Is Related To Parental Satisfaction After Single-Event Multilevel Surgery In Children With Cerebral Palsy	Journal Of Pediatric Orthopaedics	20
Haumont	2013	Flexed-Knee Gait In Children With Cerebral Palsy: A 10-Year Follow-Up Study	Journal Of Childrens Orthopaedics	19
Rutz	2013	Are Results After Single-Event Multilevel Surgery In Cerebral Palsy Durable?	Clinical Orthopaedics And Related Research	19
Ancillao	2017	Analysis Of Gait Patterns Pre- And Post-Single Event Multilevel Surgery In Children With Cerebral Palsy By Means Of Offset-Wise Movement Analysis Profile And Linear Fit Method	Human Movement Science	18
Svehlik	2016	Predictors Of Outcome After Single-Event Multilevel Surgery In Children With Cerebral Palsy	Bone & Joint Journal	18
Amirmudin	2019	Multilevel Surgery For Children With Cerebral Palsy: A Meta-Analysis	Pediatrics	17
Akerstedt	2010	Evaluation Of Single Event Multilevel Surgery And Rehabilitation In Children And Youth With Cerebral Palsy - A 2-Year Follow-Up Study	Disability And Rehabilitation	17
Khan	2007	Outcome Of Single-Event Multilevel Surgery In Untreated Cerebral Palsy In A Developing Country	Journal Of Bone And Joint Surgery-British Volume	17
Gannotti	2007	Postoperative Gait Velocity And Mean Knee Flexion In Stance Of Ambulatory Children With Spastic Diplegia Four Years Or More After Multilevel Surgery	Journal Of Pediatric Orthopaedics	17
Rutz	2012	Multilevel Surgery Improves Gait In Spastic Hemiplegia But Does Not Resolve Hip Dysplasia	Clinical Orthopaedics And Related Research	15
Edwards	2018	Predictors Affecting Outcome After Single-Event Multilevel Surgery In Children With Cerebral Palsy: A Systematic Review	Developmental Medicine And Child Neurology	14

Dequeker	2018	Evolution Of Self-Care And Functional Mobility After Single-Event Multilevel Surgery In Children And Adolescents With Spastic Diplegic Cerebral Palsy	Developmental Medicine And Child Neurology	14
Terjesen	2015	Gait Improvement Surgery In Ambulatory Children With Diplegic Cerebral Palsy A 5-Year Follow-Up Study Of 34 Children	Acta Orthopaedica	14
Gupta	2008	Single-Stage Multilevel Soft-Tissue Surgery In The Lower Limbs With Spastic Cerebral Palsy: Experience From A Rehabilitation Unit	Indian Journal Of Orthopaedics	14
Nicholson	2018	Gait Analysis Parameters And Walking Activity Pre- And Postoperatively In Children With Cerebral Palsy	Pediatric Physical Therapy	13
Galarraga	2017	Predicting Postoperative Gait In Cerebral Palsy	Gait & Posture	13
Marconi	2014	Mechanical Work And Energy Consumption In Children With Cerebral Palsy After Single-Event Multilevel Surgery	Gait & Posture	13
Chung	2008	Residual Pelvic Rotation After Single-Event Multilevel Surgery In Spastic Hemiplegia	Journal Of Bone And Joint Surgery-British Volume	13
Lofterod	2010	Changes In Lower Limb Rotation After Soft Tissue Surgery In Spastic Diplegia	Acta Orthopaedica	12
Chang	2017	Gross Motor Function Change After Multilevel Soft Tissue Release In Children With Cerebral Palsy	Biomedical Journal	11
Rajagopal	2018	Estimating The Effect Size Of Surgery To Improve Walking In Children With Cerebral Palsy From Retrospective Observational Clinical Data	Scientific Reports	10
Church	2018	Flexed-Knee Gait In Children With Cerebral Palsy A Long-Term Follow-Up Study	Bone & Joint Journal	10
Himpens	2013	Quality Of Life In Youngsters With Cerebral Palsy After Single-Event Multilevel Surgery	European Journal Of Paediatric Neurology	10
Bernthal	2010	Static And Dynamic Gait Parameters Before And After Multilevel Soft Tissue Surgery In Ambulating Children With Cerebral Palsy	Journal Of Pediatric Orthopaedics	10
Klotz	2013	Reduction In Primary Genu Recurvatum Gait After Aponeurotic Calf Muscle Lengthening During Multilevel Surgery	Research In Developmental Disabilities	9
Zwick	2001	Single Event Multilevel Surgery To Improve Gait In Diplegic Cerebral Palsy - A Prospective Controlled Trial	Zeitschrift Fur Orthopadie Und Ihre Grenzgebiete	9
Schranz	2017	Does Unilateral Single-Event Multilevel Surgery Improve Gait In Children With Spastic Hemiplegia? A Retrospective Analysis Of A Long-Term Follow-Up	Gait & Posture	8
Lehtonen	2015	Does Single-Event Multilevel Surgery Enhance Physical Functioning In The Real-Life Environment In Children And Adolescents With Cerebral Palsy (Cp)?: Patient Perceptions Five Years After Surgery	Gait & Posture	8
Zwick	2012	Does Gender Influence The Long-Term Outcome Of Single-Event Multilevel Surgery In Spastic Cerebral Palsy?	Journal Of Pediatric Orthopaedics-Part B	8
Ganjwala	2011	Multilevel Orthopedic Surgery For Crouch Gait In Cerebral Palsy: An Evaluation Using Functional Mobility And Energy Cost	Indian Journal Of Orthopaedics	8
Edwards	2020	What Is The Functional Mobility And Quality Of Life In Patients With Cerebral Palsy Following Single-Event Multilevel Surgery?	Journal Of Childrens Orthopaedics	7

Georgiadis	2017	Team Approach: Single-Event Multilevel Surgery In Ambulatory Patients With Cerebral Palsy	Jbjs Reviews	7
Park	2009	Issues Of Concern After A Single-Event Multilevel Surgery In Ambulatory Children With Cerebral Palsy	Journal Of Pediatric Orthopaedics	7
van Drongelen	2013	Gait Patterns In Twins With Cerebral Palsy: Similarities And Development Over Time After Multilevel Surgery	Research In Developmental Disabilities	6
Visscher	2022	Long-Term Follow-Up After Multilevel Surgery In Cerebral Palsy	Archives Of Orthopaedic And Trauma Surgery	4
Jones	2020	Long-Term Outcomes Following Multilevel Surgery In Cerebral Palsy	Journal Of Pediatric Orthopaedics	4
Van Rossom	2020	Single-Event Multilevel Surgery, But Not Botulinum Toxin Injections Normalize Joint Loading In Cerebral Palsy Patients	Clinical Biomechanics	4
Bishay	2013	Single-Event Multilevel Acute Total Correction Of Complex Equinocavovarus Deformity In Skeletally Mature Patients With Spastic Cerebral Palsy Hemiparesis	Journal Of Foot & Ankle Surgery	4
Koca	2011	Outcomes Of Multilevel Orthopedic Surgery In Children With Cerebral Palsy	Eklem Hastaliklari Ve Cerrahisi-Joint Diseases And Related Surgery	4
Mahmudov	2015	Comparison Of Single Event Vs Multiple Event Soft Tissue Surgeries In The Lower Extremities With Cerebral Palsy	Journal Of Orthopaedics	3
Aslan	2019	Comparison Of Single Event Multilevel Surgery And Multiple Surgical Events In The Lower Extremities Of Children With Spastic Cerebral Palsy	Eklem Hastaliklari Ve Cerrahisi-Joint Diseases And Related Surgery	2
Kin-Wah	2018	Soft Tissue Release And Osteotomies In The Treatment Of Patients With Spastic Diplegic Cerebral Palsy	Journal Of Orthopaedics Trauma And Rehabilitation	2
Steppacher	2018	Retrospective Evaluation Of Changes In Gait Patterns In Children And Adolescents With Cerebral Palsy After Multilevel Surgery	Journal Of Child Neurology	2
Lennon	2023	Is Standing Function Improved After Orthopaedic Surgery In Children With Cerebral Palsy At Gmfcs Levels Iii/Iv?	Journal Of Pediatric Orthopaedics	1
Ounpuu	2022	Cost Savings For Single Event Multilevel Surgery In Comparison To Sequential Surgery In Ambulatory Children With Cerebral Palsy	Gait & Posture	1
Min	2020	Factors Affecting Gdi Improvement After Single Event Multilevel Surgery In Patients With Cerebral Palsy	Gait & Posture	1
Wick	2018	Single-Event Multilevel Surgery To Correct Movement Disorders In Children With Cerebral Palsy	Aorn Journal	1
Martinez Caballero	2013	Multilevel Surgery For Gait Disorders In Cerebral Palsy. Quantitative, Functional And Satisfaction Outcomes Measurement	Trauma-Spain	1
Bonnefoy-Mazure	2023	A 10.5-Year Follow-Up Of Walking With Unilateral Spastic Cerebral Palsy	Journal Of Childrens Orthopaedics	0
Lennon	2023	Factors Associated With Short-Term Recovery Following Single-Event Multilevel Surgery For Children With Cerebral Palsy	Pediatric Physical Therapy	0

Moisan	2022	Assessment Of Gait Quality And Efficiency After Undergoing A Single-Event Multilevel Surgery In Children With Cerebral Palsy Presenting An Intoeing Gait Pattern	Childs Nervous System	0
Church	2022	Walking Activity After Multilevel Orthopedic Surgery In Children With Cerebral Palsy	Developmental Medicine And Child Neurology	0
Li	2021	Evaluation Of Multilevel Surgeries In Children With Spastic Cerebral Palsy Based On Surface Electromyography	Frontiers In Neuroscience	0
Nahm	2021	Single-Event Multilevel Surgery In Cerebral Palsy Value Added By A Co-Surgeon	Medicine	0
de Freitas Guardini	2021	Factors Related To Better Outcomes After Single-Event Multilevel Surgery (Semls) In Patients With Cerebral Palsy	Gait & Posture	0
Edwards	2021	A Comparison Of Conventional And Minimally Invasive Multilevel Surgery For Children With Diplegic Cerebral Palsy	Bone & Joint Journal	0
Baldwin	2020	Adding Value In Single-Event Multilevel Surgery (Semls) For Cerebral Palsy Patients With Crouch Gait: A Retrospective Study Showing Value Added By A Second Surgeon	Current Orthopaedic Practice	0
Chung	2020	Surgical Outcomes After Single Event Multilevel Surgery In Cerebral Palsy Patients With Mid-Stance Knee Hyperextension	Gait & Posture	0
Benn	2015	Multilevel Soft Tissue With Bony Corrective Surgery In Lower Limb Deformities As One Sitting Procedure In Spastic Cerebral Palsy: An Experience From Free Disabled Surgical Camps	Journal Of Evolution Of Medical And Dental Sciences-Jemds	0
